# Contact guidance persists under myosin inhibition due to the local alignment of adhesions and individual protrusions

**DOI:** 10.1038/s41598-017-14745-7

**Published:** 2017-10-30

**Authors:** Kristopher E. Kubow, Victoria D. Shuklis, Dominic J. Sales, A. Rick Horwitz

**Affiliations:** 1 Department of Biology, James Madison University, Harrisonburg, VA USA; 2 Allen Institute for Cell Science, Seattle, WA USA; 3 Department of Cell Biology, University of Virginia, Charlottesville, VA USA

## Abstract

Contact guidance—cell polarization by anisotropic substrate features—is integral to numerous physiological processes; however the complexities of its regulation are only beginning to be discovered. In particular, cells polarize to anisotropic features under non-muscle myosin II (MII) inhibition, despite MII ordinarily being essential for polarized cell migration. Here, we investigate the ability of cells to sense and respond to fiber alignment in the absence of MII activity. We find that contact guidance is determined at the level of individual protrusions, which are individually guided by local fiber orientation, independent of MII. Protrusion stability and persistence are functions of adhesion lifetime, which depends on fiber orientation. Under MII inhibition, adhesion lifetime no longer depends on fiber orientation; however the ability of protrusions to form closely spaced adhesions sequentially without having to skip over gaps in adhesive area, biases protrusion formation along fibers. The co-alignment of multiple protrusions polarizes the entire cell; if the fibers are not aligned, contact guidance of individual protrusions still occurs, but does not produce overall cell polarization. These results describe how aligned features polarize a cell independently of MII and demonstrate how cellular contact guidance is built on the local alignment of adhesions and individual protrusions.

## Introduction

Directed cell migration is an important element of numerous physiological processes including cancer metastasis, inflammation, and wound healing, as well as a critical parameter in the design of engineered tissues for regenerative medicine^1–4^. Cells determine their migration direction based on one or a combination of extracellular guidance cues, including chemical gradients (chemotaxis), adhesion gradients (haptotaxis), stiffness gradients (durotaxis), cell-cell contacts (collective cell migration; contact inhibition), and anisotropic physical features (contact guidance). Contact guidance—the tendency of cells to migrate along physical features such as grooves, aligned fibers, and substrate edges—has long been recognized as an important cue for cell migration *in vivo* ^5–8^ that is not provided by traditional *in vitro* cell culture dishes^9^. In contrast to flat, isotropic glass and plastic substrates, tissues and their mimetics provide an abundance of features that can stimulate contact guidance. For example, tumor cells in an orthotopic mammary gland mouse model orient to blood vessels and show increased invasiveness relative to cells in microenvironments with few blood vessels^10^. While cells might orient to any number of anisotropic tissue features, the fibers that comprise the tissue or scaffold are of special interest, because cells have the ability to reorganize them and create their own contact guidance features. One of the most common observations of contact guidance in 3D fibrillar environments is that cells apply force to the fibers, causing them to align, and then migrate along these “tracks” (e.g. refs^11–15^). This general phenomenon has been shown both *in vivo* and *in vitro* to be involved in guiding mammary epithelial branching direction^16^ and in facilitating tumor cell invasion into the surrounding tissue^17,18^.

Extensive research using reductionist cell culture models such as gratings and microcontact printed lines of extracellular matrix (ECM) proteins, as well as biomimetic 2D and 3D systems has led to the formation of two general, non-mutually-exclusive hypotheses about the biological mechanisms underlying contact guidance^2,4,19^. Substrates with large spacings between aligned features prevent cells from spreading across multiple ridges, fibers, or adhesive lines, thereby enforcing contact guidance along the one or two features that can be contacted^8,19,20^. More applicable to cells migrating in dense tissue where potential contact guidance features abound is the focal adhesion restriction theory first proposed by Ohara and Buck^7^. According to this hypothesis, ECM fibers and features of similar dimensions (e.g. thin ridges), provide an essentially one-dimensional substrate upon which adhesions can only elongate and mature in one direction^19–24^. Because adhesions grow linearly, those elongating in the direction of fiber alignment have a large area on which to grow, while those elongating perpendicularly are restricted to the width of the fiber (typically < 1 µm). This dichotomy results in differences in adhesion composition^22^ and actomyosin organization^20,22^, leading to cell polarization in the direction of feature alignment. When the aligned features are deformable (e.g. aligned fibrillar collagen matrices), contact guidance is also likely influenced by anisotropic substrate stiffness. Adhesions oriented along the long axis of aligned fibers sense a greater stiffness than those oriented perpendicularly^25,26^. Thus, contact guidance in ECM appears to involve elements of durotaxis and haptotaxis because aligned fibers provide both greater stiffness and greater co-linear adhesive area than randomly oriented fibers.

Migration guidance cues, whether chemical or physical, operate by polarizing a cell’s cytoskeleton to generate a protrusive front and a non-protrusive and/or contractile rear that directs the cell in a specific direction relative to the cue^1^. In cells that exhibit adhesion-based crawling migration (e.g. fibroblasts, branching endothelial cells), non-muscle myosin IIB (MIIB) stabilizes adhesions and locally inhibits protrusive signaling thereby defining the cell rear, while a zone of intense actin polymerization, opposite the rear, characterizes the front^22,27–29^. Cells on microcontact printed adhesive lines, 2D arrays of aligned fibers, and in 3D fibrillar ECMs organize non-muscle myosin (MII) along their lateral edges, focusing protrusive activity in the direction of substrate feature alignment^22,27,29–31^. Interestingly, while MII contractility is necessary to align randomly oriented fibers, there are reports that MII is not necessary to polarize cell migration on pre-aligned features^16,23,31–33^, although the extent of the polarization may be reduced^20,21,24,34–37^. Given that MII organization and activity are integral to adhesion maturation and durotaxis^38,39^—the hypothesized bases for contact guidance in ECM—and localizing cell protrusive activity, it is unclear how contact guidance is maintained under MII inhibition.

In this study, we use 2D fibrillar substrates and 3D collagen ECMs to investigate the ability of cells to sense and respond to fiber alignment in the absence of MII organization and contractility. We focus here on the aspects of contact guidance related to the establishment of a polarized cell morphology and the polarization of cell protrusions, which are the elements that ultimately determine migratory persistence^28,38^. We find that contact guidance occurs at the level of individual protrusions, which are guided by local fiber orientation independently of one another and independent of MII activity. Individual protrusion stability and persistence are a function of adhesion lifetime, which, in agreement with previous work, depends on fiber orientation. However, when MII activity is inhibited and adhesion lifetime no longer depends on fiber orientation, protrusion stability and persistence appear to depend on the ease with which protrusions can continue to form adhesions in a given direction. The ability to form closely spaced adhesions sequentially, without having to skip over gaps in adhesive area, biases protrusion formation in the direction of fiber alignment. Therefore, while MII activity controls the polarity of protrusion initiation by establishing non-protrusive zones^22,28,29^, it is not essential for biasing protrusion stability in the direction of feature alignment and therefore not essential for contact guidance.

## Results

### Cells polarize to aligned fibers regardless of MII activity

Based on our current understanding, MII activity is involved in two critical aspects of cell polarity determination. First, MII creates non-protrusive zones along the edges of cells on 2D substrates and in 3D matrices, and its inhibition causes cells to protrude indiscriminately in multiple directions, thereby reducing cell polarity^22,27–29^. Second, MII activity is hypothesized to be instrumental in contact guidance because of its role in adhesion maturation^20–22,34^. MII inhibition does eliminate the polarity of cells on isotropic substrates; however, in contrast to its hypothesized roles, it does not necessarily eliminate the polarity of cells on substrates with aligned features^16,23,31–33^.

**Figure 1:**
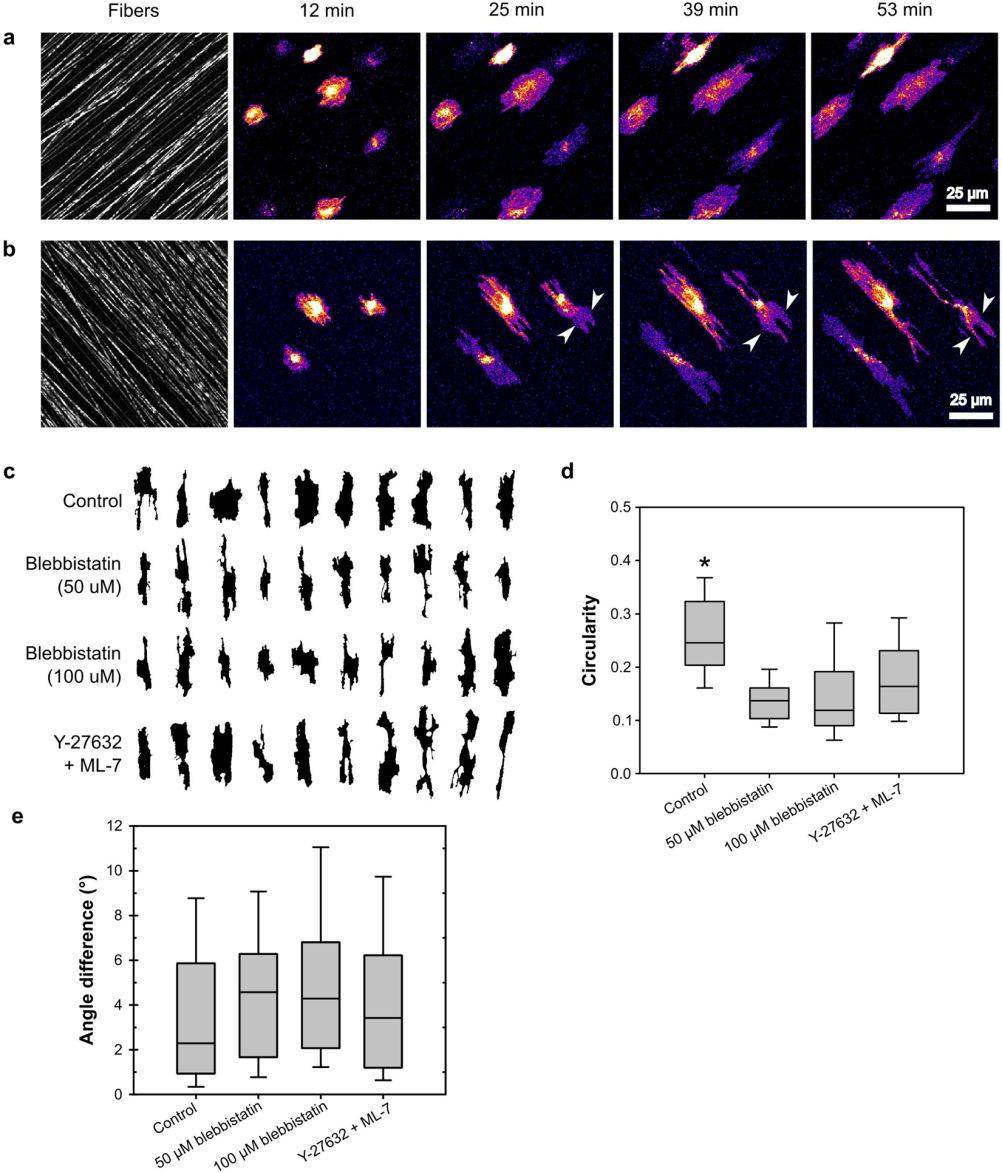
HT-1080 cells orient to aligned fibers even under MII activity and organization inhibition. HT-1080 cells, stained with the membrane dye DiI were seeded on 2D PCL scaffolds with aligned fibers. (**a** and **b**) Selected frames from Supplementary Movies 1 and 2, respectively. Cells were imaged under control conditions (**a**) or treated with a combination of 20 µM Y-27632 and 10 µM ML-7 (**b**). Cells (DiI stain) are displayed using an intensity-based heatmap to better visualize them in spite of their large differences in brightness. The color of the heatmap has no physiological relevance. Under both control and inhibited conditions, cells protruded along the fibers, resulting in overall cell alignment to the fibers. Although protrusions perpendicular to the direction of fiber alignment did occur (often during spreading), the protrusions subsequently turned and proceeded along the fibers (e.g. arrowheads in B). Scale bar, 25 µm. (**c–e**) Images of multiple cells under control or inhibited conditions were acquired, 30 min after seeding: control, 53 cells from three experiments; 50 µM blebbistatin, 48 cells from two experiments; 100 µM blebbistatin, 44 cells from two experiments; Y-27632/ML-7, 33 cells from two experiments. (**c**) Representative silhouettes of cells illustrating their morphology and orientation under control conditions, treated with blebbistatin (50 and 100 μM), and treated with Y-27632/ML-7. All silhouettes have been rotated so that the substrate fibers (not depicted) in each image are aligned vertically. Cells and their individual protrusions remained aligned to fibers under all conditions. (**d**) Plot of the “circularity” morphological parameter. Center line of each box indicates the median; upper and lower bounds of the box indicate the 75^th^ and 25^th^ percentiles, respectively; the “whiskers” indicate the 10^th^ and 90^th^ percentiles. Inhibited cells had a significantly lower circularity than control cells (p < 0.01, Kruskal-Wallis test, Tukey-Kramer posthoc), indicating that they exhibited a higher number of protrusions^22^. (**e**) Plot of the difference in angle between cell orientation and the average direction of fiber alignment. All groups showed a median deviation of less than 5 degrees and a deviation of less than 11 degrees for at least 90% of their individuals.

To better understand the roles of MII activity and adhesion maturation in contact guidance, we measured the effects of MII inhibition on cell protrusion and polarity on aligned substrates. We used 2D scaffolds composed of aligned electrospun polycaprolactone (PCL) fibers, which mimicked the fibrous nature of ECM, but were sufficiently stiff to prevent nearly all cell-mediated fiber reorganization^22^. HT-1080 human fibrosarcoma cells seeded onto fibronectin-adsorbed, aligned PCL scaffolds exhibited contact guidance, elongating and migrating in the direction of fiber orientation (Fig. 1a, Supplementary Movie 1). Unlike cells on isolated single linear features^30,32^, the cells spanned multiple neighboring fibers similar to closely spaced adhesive lines and gratings^21,31,35^. During spreading, cells protruded in all directions, including perpendicular to the direction of fiber alignment as observed previously^21,31,35^. However, once spread, the protrusive activity continued primarily along the fiber, parallel to the direction of fiber orientation (Fig. 1a). Cells incubated with a combination of the Rho kinase (ROCK) inhibitor Y-27632 and the MII light chain kinase (MLCK) inhibitor ML-7 exhibited a greater number of protrusions than control cells, but still showed persistent protrusion in the direction of fiber alignment (Fig. 1b, Supplementary Movie 2). Even after spreading, protrusions sometimes occurred perpendicular to the fibers; however these protrusions either retracted or turned 90° and protruded along the fiber (Fig. 1b, arrowheads).

To quantify cell morphology and orientation, we analyzed silhouettes of cells that were imaged 30 min after seeding and performed a semi-automated morphological analysis. Figure 1c shows representative cell silhouettes under different experimental conditions, reoriented so that the direction of fiber alignment is vertical. Cells treated with Y-27632/ML-7 or with the MII ATPase inhibitor blebbistatin were more protrusive than control cells as quantified by the circularity morphological parameter (Fig. 1d) and as expected based on the known role of MII in restricting protrusion^28^ and previous reports^31^. However, although the inhibitor-treated cells had more protrusions, nearly all of these protrusions extended in the direction of fiber alignment (Fig. 1b,c). Indeed, 80% of cells treated with 50 μM blebbistatin were oriented to within 10° of the average fiber orientation (Fig. 1e). Doubling the blebbistatin concentration yielded similar results (Fig. 1c,d,e), as did experiments utilizing NIH 3T3 fibroblasts (Supplementary Fig. S1). Therefore, although MII activity inhibition decreased the ability of cells to restrict the number and direction of their protrusions, these protrusions individually polarized along fibers, thereby allowing the cells to orient in the direction of fiber alignment.

### Cells polarize to aligned fibers even when adhesion lifetime is uniformly minimized

The hypothesized mechanism for contact guidance is based on differences in the maturation of adhesions formed along the long axis of vs. oblique to an aligned feature. Adhesions along aligned fibers should exhibit greater growth and longer lifetimes, leading to increased protrusion stability and persistence^20–22,34^. However, our observations show that individual protrusions exhibited contact guidance even in the absence of MII activity. To determine the basis for these observations, we analyzed adhesion lifetimes of cells on aligned fibers under normal conditions and under MII inhibition.

**Figure 2:**
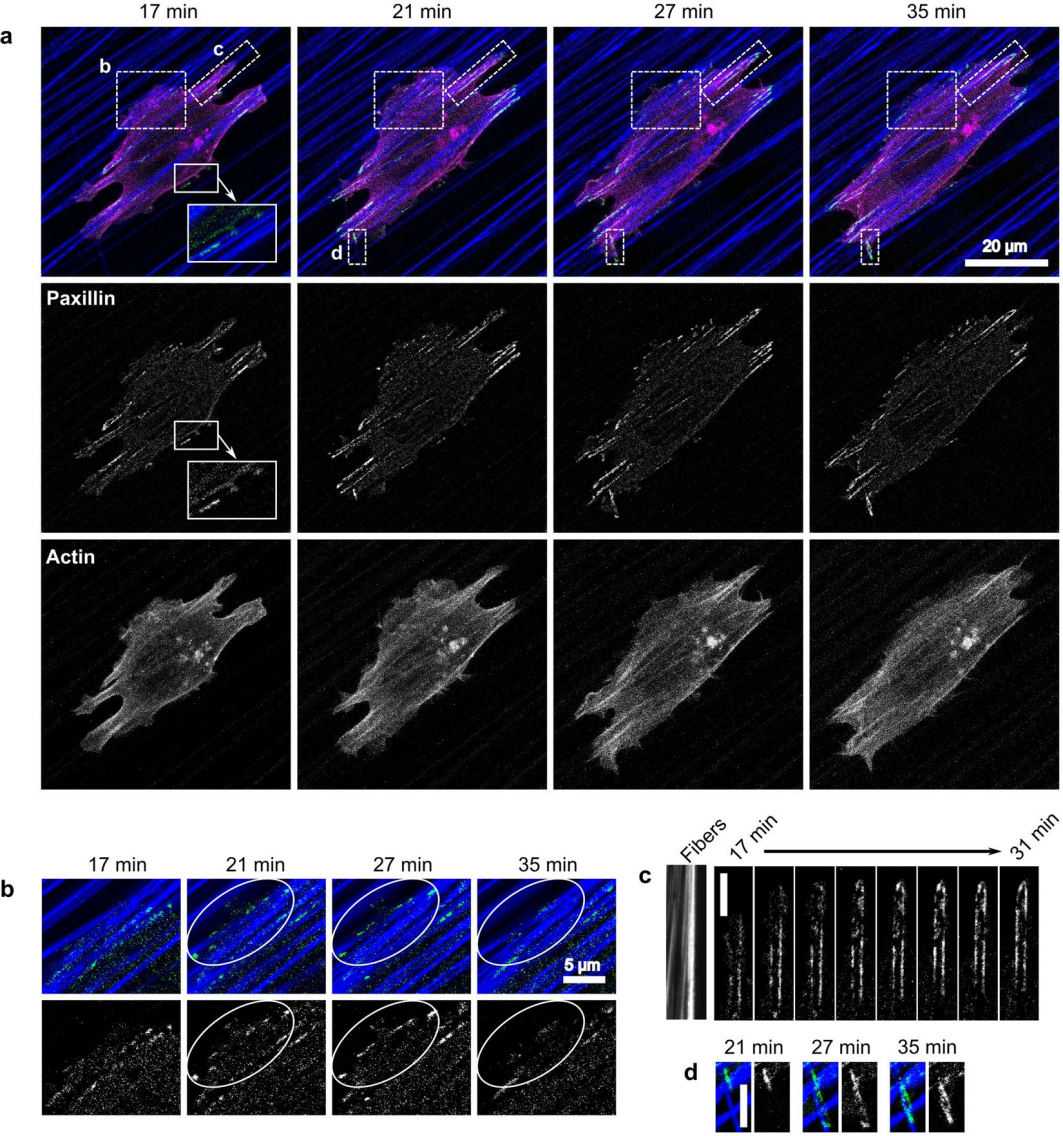
The persistence of individual protrusions is correlated with adhesion stability and fiber orientation. HT-1080 cells expressing EGFP-paxillin (green) and Ruby-Lifeact (actin; magenta) were seeded on 2D PCL scaffolds with aligned fibers (blue) and imaged over time. Note that small fluctuations in focus changed the intensity of the reflectance signal coming from the fibers, resulting in a few fibers appearing very dim in some frames. Times indicated in the figure are relative to the time of seeding. All images are maximum projections of z-stacks. Paxillin images have been additionally processed (“flattened”) to reduce background noise (see Materials and Methods). (**a**) Selected frames from Supplementary Movie 3, representative of seven independent experiments. The cell forms adhesions primarily along aligned fibers (e.g. panels C and D) but also in protrusions perpendicular to the direction of alignment (e.g. panel B and inset at 17 min). (**b**) Enlargement of area B in panel A, showing a protrusion that has formed perpendicular to the direction of fiber alignment. The protrusion extends from 17 to 21 min and forms small adhesions (see area in white oval), which quickly disappear when the protrusion is retracted beginning at 27 min. (**c**) Enlargement of area C in panel A, showing a protrusion in the direction of fiber alignment. The protrusion forms adhesions as it progresses along the fibers. (**d**) Enlargement of area D in panel A. A protrusion extending in the direction of fiber alignment encounters a fiber perpendicular to its path (at 21 min in panel A) and splits, sending a second protrusion along the angled fiber. This secondary protrusion forms large, stable adhesions as it progresses along the fiber. Scale bars: 20 µm (**a**), 5 µm (**b**,**c**,**d**).

HT-1080 cells were co-transfected with Ruby-Lifeact (an actin maker^40^) and EGFP-paxillin, seeded onto aligned PCL scaffolds, and imaged over time, similar to the experiments in Fig. 1. Figure 2 shows frames from a movie of a representative cell under control conditions (no MII inhibition; Supplementary Movie 3). At the beginning of imaging, the cell was already spread and polarized in the direction of fiber orientation. The primary protrusions, in front of and behind the cell contained numerous large adhesions. There were also smaller protrusions extending perpendicular to the fibers and containing small adhesions (17 min, Fig. 2a inset and 2b). Over time, the primary protrusions proceeded along the fibers, with adhesions progressively forming in front of one another (Fig. 2c). The broad protrusion extending perpendicular to the fibers (Fig. 2b) protruded up through 21 min and formed multiple small adhesions; however the protrusion retracted and most of the adhesions disappeared by 35 min. Interestingly, the cell encountered a single fiber that was roughly perpendicular to the predominant alignment (Fig. 2d) and began following this fiber, forming stable protrusions and adhesions.

The two contrasting cases of protrusions perpendicular to the cell orientation (Fig. 2b and d) implicate adhesions in directing the polarity of stable protrusions. The protrusion that extended perpendicular to the fibers (Fig. 2b) formed multiple small, but no large, adhesions and was eventually retracted by the cell. The protrusion that extended along a single, errantly oriented fiber (Fig. 2d) formed stable adhesions and persisted through the end of the movie. The protrusion that extended across the fibers was likely retracted through MII-mediated “pruning”^29^. The protrusion that extended along a fiber was also at a high angle to the cell cytoskeleton (Fig. 2a, magenta and bottom row), yet it persisted, perhaps because it was able to form more stable adhesions.

**Figure 3:**
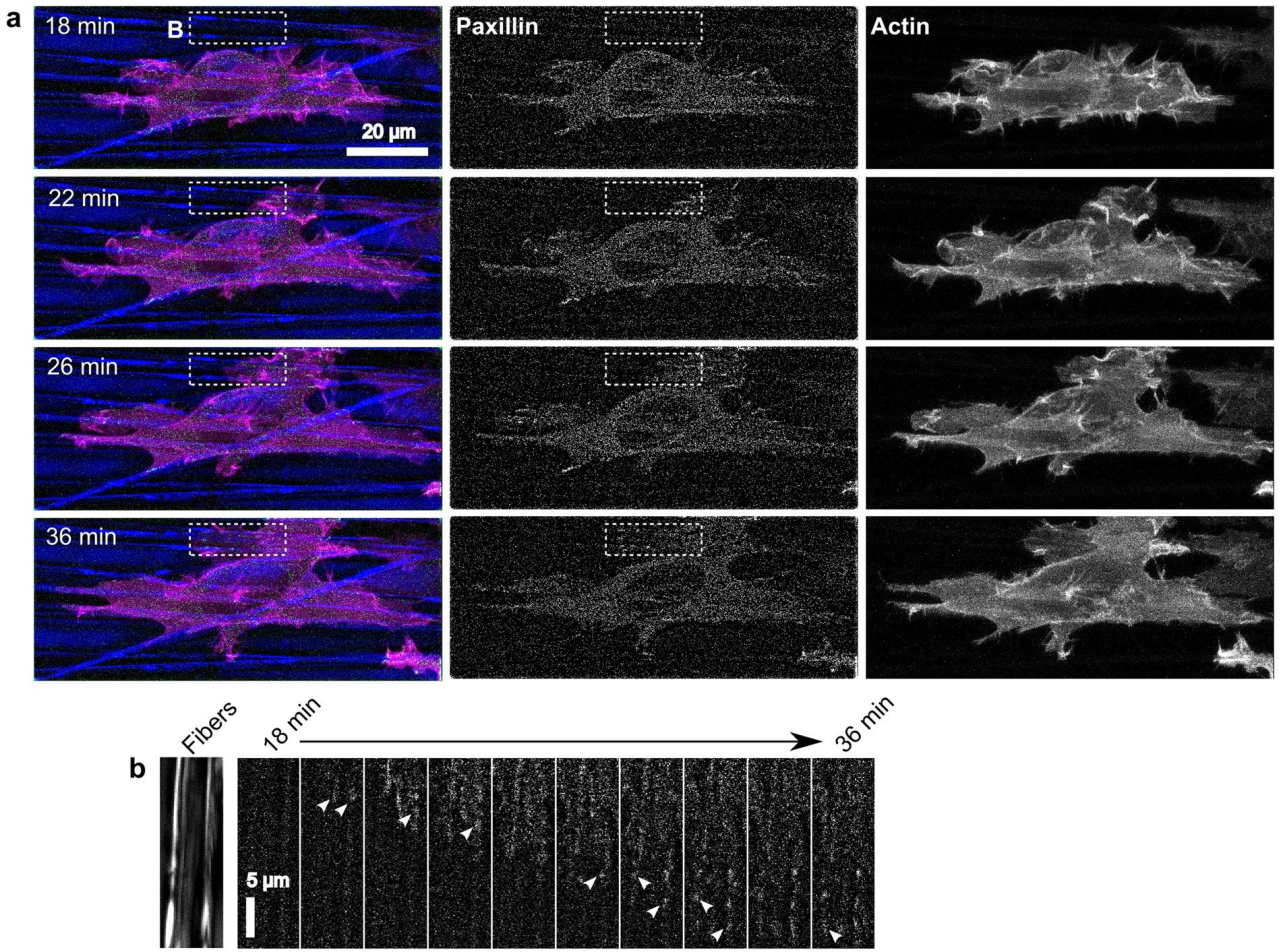
The persistence of individual protrusions is correlated with fiber orientation even when under MII inhibition. HT-1080 cells expressing EGFP-paxillin (green) and Ruby-Lifeact (actin; magenta) were seeded on 2D PCL scaffolds with aligned fibers (blue) in the presence of 20 µM Y-27632 and 10 µM ML-7 and imaged over time. Times indicated in the figure are relative to the time of seeding. All images are maximum projections of z-stacks. Paxillin images have been additionally processed (“flattened”) to reduce background noise (see Materials and Methods). (**a**) Selected frames from Supplementary Movie 4 representative of seven independent experiments. The cell aligns to the predominant direction of fiber orientation, despite it being highly protrusive in nearly all directions. Adhesions (center column of images) are generally small. (**b**) The cell forms a protrusion perpendicular to the direction of fiber alignment shortly after 18 min, then turns approximately 90 degrees and protrudes along the aligned fibers, progressively forming small transient adhesions (arrowheads) as it extends. Scale bars: 20 µm (**a**), 5 µm (**b**).

These observations support the mechanism whereby fiber orientation controls protrusion stability by regulating adhesion lifetime. However, adhesion maturation and lifetime depend on MII activity^38^ and our previous experiments indicated that fiber orientation controls cell orientation even in under MII inhibition. We therefore repeated the above experiment in the presence of a cocktail of Y-27632 and ML-7, which produce similar effects to blebbistatin on cell morphology and adhesion (Fig. 1 and ref.^22^). Blebbistatin was not used because it is incompatible with live cell imaging using blue light (e.g. 488 nm laser used to excite EGFP-paxillin)^41^. As expected, cells treated with the inhibitors (Fig. 3a, Supplementary Movie 4) were more protrusive and had fewer and smaller visible adhesions than in control cells (Fig. 2a). Nevertheless, as seen in earlier experiments (Fig. 1), the cells remained oriented in the direction of fiber alignment. Stable protrusions that extended perpendicular to the alignment axis, turned and protruded along the newly contacted fibers (Fig. 3b) similar to our previous observations (Fig. 1b). This agrees with a previous report that blebbistatin reduced adhesion size in cells on isolated polymer fibers, but did not affect cell alignment^23^.

**Figure 4:**
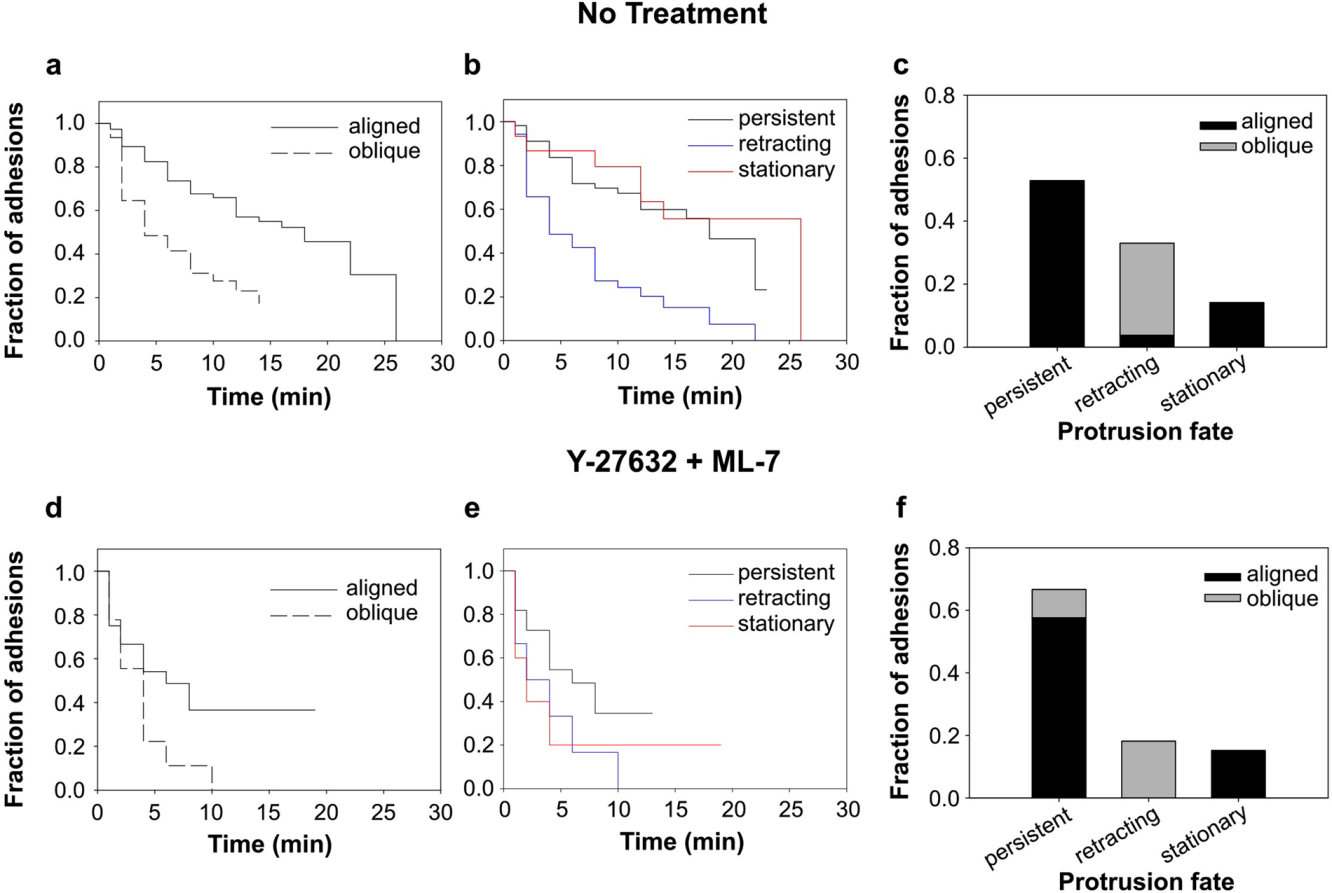
Persistent protrusions occur predominantly along aligned fibers even when adhesion lifetime is reduced. Quantitative analysis of the 14 movies represented by Figs 2 and 3. Movies of HT-1080 cells on 2D PCL scaffolds with aligned fibers under control (**a**–c) conditions or in the presence of 20 µM Y-27632 and 10 µM ML-7 (**d–f**) were analyzed with respect to adhesion lifetime, protrusion fate, and fiber orientation. Due to the highly aligned PCL fiber substrate, protrusions were nearly always perfectly aligned to fibers or at high angles (approaching 90°). Because of this and the inherent uncertainty in identifying precise fiber and protrusion angles, we classified protrusions as either “aligned” or “oblique” (see Materials and Methods). (**a**,**d**) Survival curves for adhesions associated with aligned and oblique fibers. Under control conditions, adhesions on aligned fibers had longer median lifetimes than those at oblique angles (16.7 vs. 3.8 min; p < 0.001 log-rank test). MII inhibition reduced the median lifetime of adhesions on aligned fibers from 16.7 to 5.5 min (p = 0.018, log-rank test). The median lifetime of adhesions to oblique fibers was reduced slightly (3.8 to 2.3 min, n.s.). Number of adhesions analyzed: control-aligned, 75; control-oblique, 31; inhibited-aligned, 24; inhibited-oblique, 9. (**b**,**e**) Survival curves for adhesions associated with persistent, retracting, and stationary protrusions. Under control conditions, the median lifetime of adhesions in retracting protrusions (3.8 min) was shorter than for adhesions in persistent and stationary protrusions (17.3 and 18.8 min, respectively; p < 0.01 log-rank test). Median lifetimes under MII inhibited conditions were not significantly different. Number of adhesions analyzed: control-persistent, 56; control-retracted, 35; control-stationary, 15; inhibited-persistent, 22; inhibited-retracted, 6; inhibited-stationary, 5. (**c,f**) Bar graph showing fraction of adhesions associated with aligned vs. oblique fibers and the three protrusion fates. For both control and MII inhibited conditions, adhesions associated with oblique fibers were also associated with retracting protrusions.

To investigate the relationship between adhesion stability, fiber angle, and protrusion stability, we tracked individual adhesions in multiple timelapse movies and quantified their lifetimes as a function of the angle of the fiber relative to the protrusion direction. As a result of the highly aligned PCL fiber substrate, protrusions were nearly always perfectly aligned to fibers or at high angles (approaching 90°). Because of this and the inherent uncertainty in identifying precise fiber and protrusion angles, we classified protrusions as either “aligned” or “oblique” (see Materials and Methods). Survival curve analysis (Fig. 4a) revealed that adhesions in protrusions aligned to fibers had longer median lifetimes than those at oblique angles (16.7 vs. 3.8 min; p < 0.001 log-rank test). The shorter lifetime of adhesions on oblique fibers was due in large part to a precipitous drop in survival within the first four minutes after formation. This low survival rate subsequently transitioned to a higher survival rate (lower slope of the survival curve), more similar to that of the adhesions on aligned fibers (Fig. 4a). The steep initial phase of the oblique survival curve reflects the tendency of protrusions on oblique fibers to retract within the first few minutes of attachment. Indeed, all of the adhesions observed on oblique fibers were associated with protrusions that retracted over the duration of filming (Fig. 4c) and the survival curve of adhesions in retracting protrusions (Fig. 4b, blue) resembled that of adhesions to oblique fibers (Fig. 4a). In contrast, nearly all adhesions to aligned fibers were associated with persistent (actively advancing) or stationary protrusions (Fig. 4b,c).

MII contractility inhibition significantly reduced the median lifetime of adhesions on aligned fibers (compare Fig. 4a,d) from 16.7 to 5.5 min (p = 0.018, log-rank test). The median lifetime of adhesions to oblique fibers was reduced slightly (3.8 to 2.3 min, not statistically significant), but the major effect of MII inhibition was to effectively eliminate the second phase of the survival curve seen under control conditions (Fig. 4a). Similar results were obtained when the data were plotted as a function of protrusion fate (Fig. 4e). The general decrease in longer-lived adhesions, regardless of fiber orientation, indicates that MII contractility plays an important role in adhesion stabilization and is responsible for much of the difference in adhesion lifetimes as a function of fiber alignment and protrusion fate that were observed in control cells (Fig. 4a,b,c). Indeed, proportionally fewer adhesions to oblique fibers were associated with retracting protrusions (Fig. 4f), presumably due to decreased MII-mediated protrusion pruning^29^. Nevertheless, although the difference in adhesion lifetimes on aligned and oblique fibers was dramatically reduced (compare Fig. 4a,d), cells continued to align to fibers (Fig. 1f) and adhesions in persistent and stationary protrusions still predominantly associated with aligned fibers (compare Fig. 4c,f). Therefore, while a contact guidance mechanism involving differential adhesion maturation and stability is sufficient to explain our observations under control conditions, our observations under MII inhibition suggest that an additional adhesion-based mechanism is involved in biasing protrusion in the direction of fiber alignment.

### Gaps between fibers provide an obstacle to sequential adhesion formation

**Figure 5:**
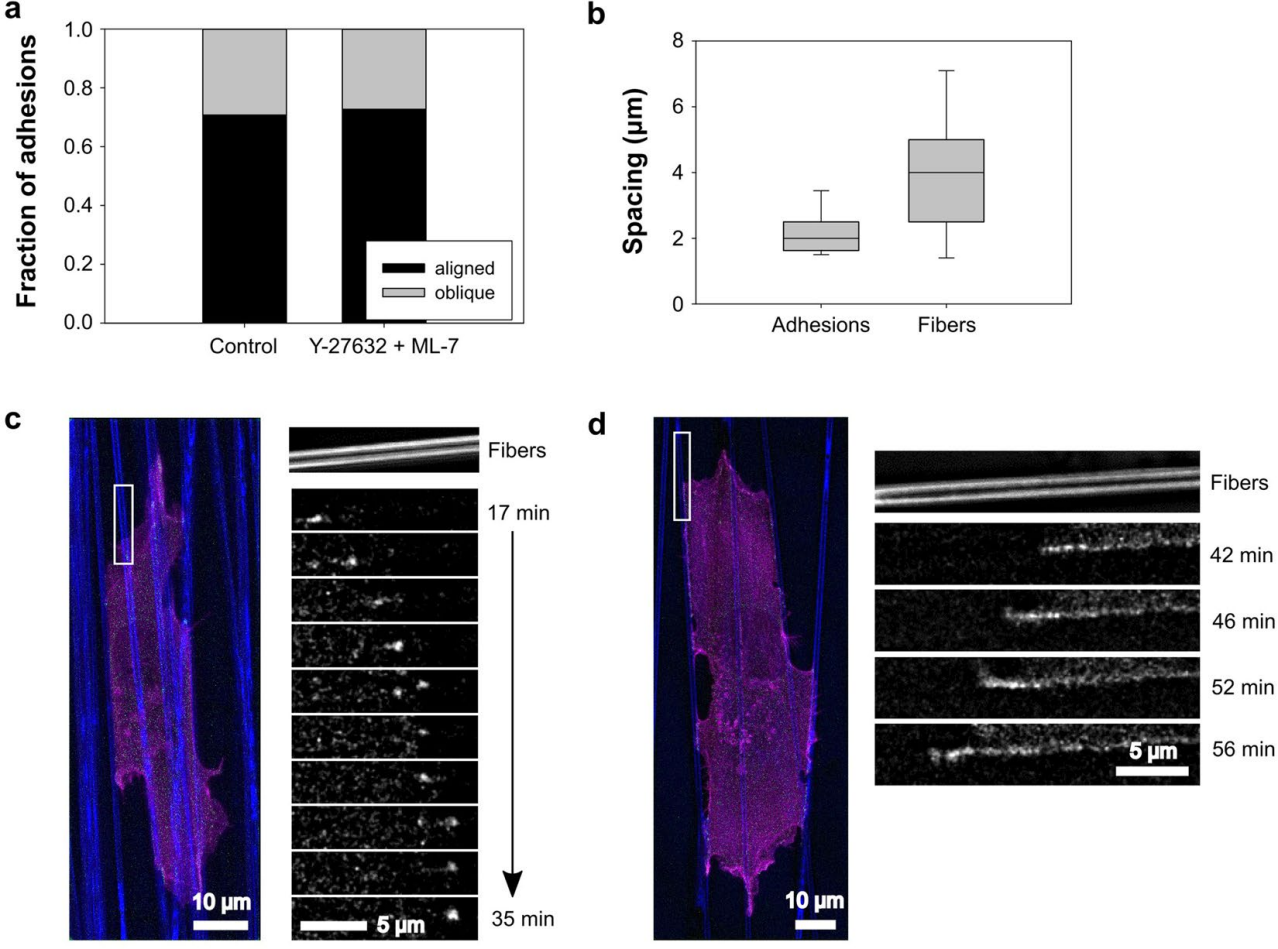
Aligned fibers provide a continuous substrate for the sequential formation of adhesions. (**a**) Fraction of new adhesions associated with protrusions aligned or oblique to the predominant fiber orientation on aligned PCL scaffolds in the presence of absence of 20 µM Y-27632 and 10 µM ML-7. Distribution of adhesions on aligned vs. oblique fibers is essentially unchanged under MII inhibition. (**b**) HT-1080 cell under MII inhibition, cultured on an aligned PCL substrate (blue) and expressing EGFP-paxillin (green) and Ruby-Lifeact (actin; magenta). Inset: Small, transient adhesions form sequentially as a protrusion progresses along a fiber (inset is rotated 90 degrees clockwise with respect to the color image). (**c**) Distribution of spacing between newly formed adhesions in HT-1080 cells under MII inhibition and between fibers in aligned PCL scaffolds (in the direction perpendicular to fiber alignment). Average spacing between PCL fibers is significantly larger than average spacing between sequentially formed adhesions (p < 0.01; Kruskal-Wallis test; n = 20 adhesions, 67 fibers). (**d**) MII-inhibited HT-1080 imaged under conditions similar to panel B. Inset: Adhesion appears to grow from distal end (left side), likely due to the sequential formation of closely spaced sub-diffraction adhesions as the protrusion advances along the fiber (inset is rotated 90 degrees counter-clockwise with respect to the color image). Images in panels B and D are representative of observations made in seven independent experiments. Data are from same experiments as Fig. 4. Times indicated in the figure are relative to the time of seeding. All images are maximum projections of z-stacks. Paxillin images have been additionally processed (“flattened”) to reduce background noise (see Materials and Methods). Scale bars, 10 µm (main panels), 5 µm (insets).

In our analysis of the above experiments, we observed that, in both control and MII-inhibited cells, adhesions formed preferentially in the direction of fiber alignment, rather than at oblique angles (Fig. 5a). Even with the significant reduction in the lifetime of adhesions on aligned fibers under MII inhibition (Fig. 4a,d), the ratio of adhesions forming on aligned vs. oblique fibers remained the same. This further suggests that higher adhesion lifetime is not the only mechanism capable of biasing adhesion formation to aligned fibers. A potential complementary mechanism is that it is simply easier for an active protrusion to form a sequential series of adhesions along the continuous surface of a fiber, rather than skipping over gaps between fibers. In support of this hypothesis, we observed the sequential formation of adhesions as protrusions extended along fibers in control (Fig. 2c) and MII-inhibited cells (Figs 3b and 5b). Similar observations have been made in cells cultured in 3D collagen ECMs^22^ and 1D fibers^30^. In MII-inhibited cells, sequentially formed adhesions were spaced an average (median) of 2 µm apart (Fig. 5c); however this is likely an overestimate because under our experimental conditions it was not possible to visualize nascent adhesions. Moreover, we also observed new adhesions being added very close to the distal ends of existing adhesions (Fig. 5d, similar to 4c) such that it was not possible to measure the spacing. In comparison, the median lateral spacing between fibers in the PCL scaffolds was 4 µm, which was greater than the spacing between 80% of measurable sequentially formed adhesions (Fig. 5c). These data suggest that the gaps between fibers can act as a barrier to the sequential formation of adhesions and therefore the persistence of their associated protrusion. Therefore, aligned features could enable contact guidance in MII-inhibited cells by providing a continuous adhesive surface that biases the progression of individual protrusions, causing the overall polarization of the cell.

### Under MII inhibition, individual protrusions exhibit contact guidance regardless of fiber orientation

**Figure 6:**
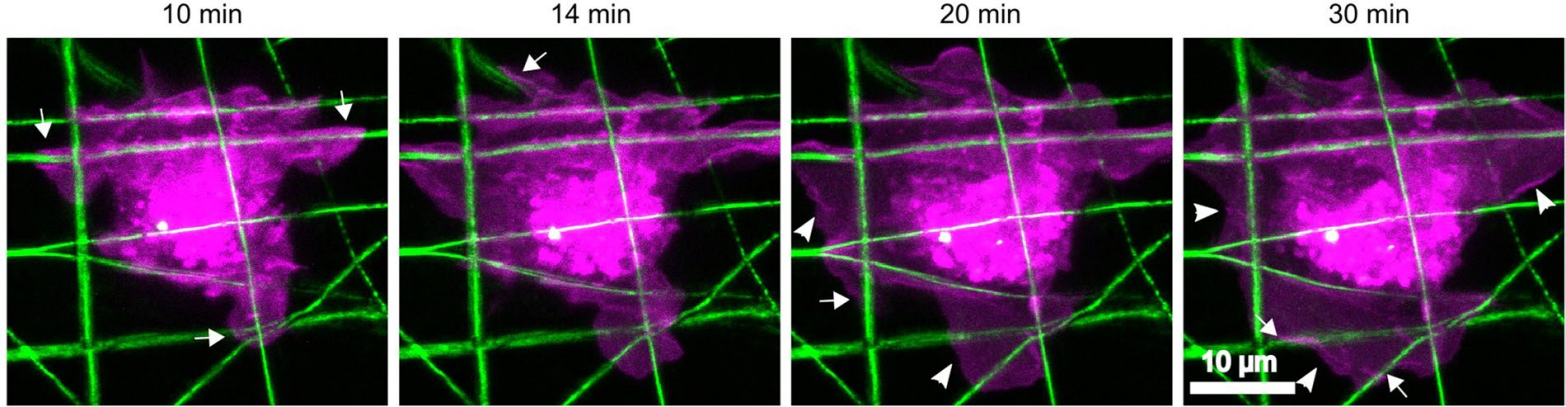
HT-1080 cell protrusions exhibit contact guidance along randomly oriented PCL scaffold fibers. HT-1080 cells, stained with the membrane dye DiI (magenta) were seeded on 2D PCL scaffolds (green) in the presence of 50 µM blebbistatin and imaged over time. Images are selected frames from Supplementary Movie 5, representative of six independent experiments (see Supplementary Fig. S2 for an additional image set). Times indicated in the figure are relative to the time of seeding. The cell protrudes in multiple directions, with each protrusion independently extending along a fiber (arrows), rather than skipping over gaps between fibers. Lamellae often stretch between protrusions that are anchored to nearby fibers (arrowheads). All images are maximum projections of z-stacks. Scale bar, 10 µm.

The above hypothetical mechanism suggests that, under MII inhibition, an individual protrusion should progress along its associated fiber, regardless of the fiber’s orientation. To test this prediction, we performed timelapse imaging of HT-1080 cells spreading on PCL scaffolds with randomly oriented fibers in the presence of 50 μM blebbistatin. The cells attached to the scaffolds within 5 min of seeding and imaging began approximately 5–25 min later. Figure 6 shows a representative cell on a PCL scaffold (Supplementary Movie 5; additional image set in Supplementary Fig. S2). The cell’s protrusions extend along fibers at various angles to the cell, following the direction of the fibers rather than skipping over them in an independent direction. This stands in contrast to cells on isotropic 2D substrates (e.g. fibronectin-adsorbed glass) in which MII inhibition by blebbistatin causes loss of polarity and indiscriminate protrusion in multiple directions^28^. While blebbistatin-treated cells on PCL scaffolds were highly protrusive and did not show an overall cell polarity, their individual protrusions followed the orientation of their associated fibers. Had the PCL fibers been aligned, the individual protrusions would have oriented in the same direction, resulting in cell polarization (Fig. 1).

While these observations support our sequential-adhesion-based contact guidance mechanism, the gaps between the PCL fibers were large relative to the gaps that typically exist between fibers in connective tissue ECM^20,42^. We therefore asked if cells would behave similarly in 3D fibrillar collagen matrices. Unlike stiff PCL fibers, collagen fibers can be moved and aligned by cells. Indeed, as adherent cells polarize and migrate in pliable, randomly oriented ECM, they will simultaneously align fibers in the direction of migration, creating a positive feedback loop between cell polarization and fiber alignment (e.g. refs^11,13,14,16–18,33^). To minimize any such “pre-conditioning” of the collagen ECM fiber structure, and therefore any associated effect on the polarization of individual cells, we investigated the earliest stages of the cell polarization process. We seeded HT-1080 cells sparsely in rat-tail collagen type I ECMs and began imaging their movements within 10 min. Rat-tail (non-pepsinized) collagen polymerizes much faster than bovine dermis collagen^42^ and a dense network of randomly oriented collagen fibers was already visible by 5 min after the onset of gelation.

**Figure 7:**
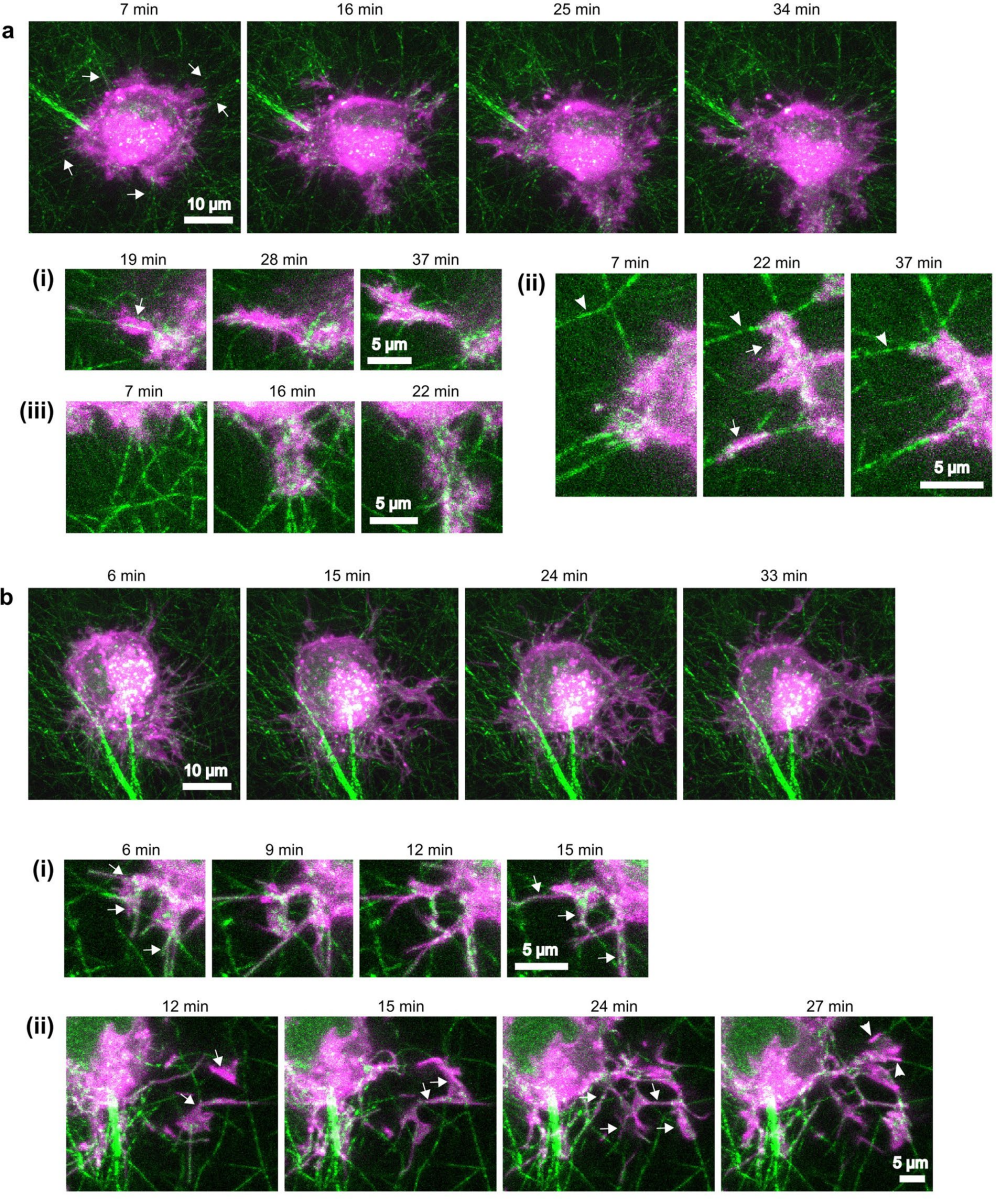
HT-1080 cell protrusions exhibit contact guidance along collagen I fibers under control or blebbistatin-treated conditions. HT-1080 cells, stained with the membrane dye DiI (magenta) were seeded in 1.2 mg/ml rat-tail collagen ECMs (green) and imaged over time. Times indicated in the figure are relative to the time of seeding. Primary panels (a and b) are maximum projections of z-stacks. The sub-panels (i,ii,iii) show selected frames and z-slices from regions of interest in the associate full-cell image. (**a**) Selected frames from Supplementary Movie 6, representative of seven independent experiments. (7–16 min) The cell initially protrudes in multiple directions. Stable protrusions coincide with centripetally aligned fibers (e.g. arrows). (16–25 min) Cell begins moving predominately in one direction (toward the bottom of the frame) and the associated protrusion/retraction activity compacts fibers and aligns them in the direction of migration (see A.iii and Supplementary Movie 6 for clearer depiction of fiber movement). **(a**.**i)** Protrusion progresses along an aligned fiber. **(a**.**ii)** Two protrusions progress along centripetally aligned fibers (arrows). The tensile forces applied during the protrusion/retraction cycles cause the movement and reduction in angle (alignment) of fibers (arrowheads). **(a**.**iii)** A protrusion causes fiber alignment and compaction. **(b)** Selected frames from Supplementary Movie 7 of a cell treated with 50 µM blebbistatin (representative of four independent experiments). The cell extends many thin and highly dynamic protrusions. Protrusions are not localized to areas of high matrix alignment, but a higher number of protrusions extend toward the bottom of the frame, possibly in response to the predominant alignment of the fibers. There is little cell-mediated fiber movement; instead, protrusions often follow fibers like tracks, even if they do not follow a straight line. **(b**.**i)** Protrusions progress along fibers at multiple angles to the cell. **(b**.**ii)** (12 min) Two protrusions appear (arrows), extending downward from a different z-plane (not depicted). (15–24 min) The two main protrusions split into multiple smaller protrusions (arrows) that progress along fibers at multiple angles to the cell. (27 min) A third main protrusion progresses along two, non-aligned fibers (arrowheads). Scale bars, 10 µm (main panels), 5 µm (sub-panels).

In control samples (no MII inhibition), we routinely observed bundles of fibers centripetally aligned to cells and terminating at small protrusions (Fig. 7a, Supplementary Movie 6). These were detected even at our earliest observable timepoints (6–7 min after seeding), suggesting that cells begin aligning fibers at the very onset of spreading. Cells exhibited cycles of protrusion along centripetally aligned fibers and retraction that applied force sufficient to enhance fiber alignment in the direction of protrusion (Fig. 7a, i-iii). These observations suggest that, even at very early timepoints, cell-generated matrix fiber alignment serves as a local polarity cue that determines the direction of individual protrusions, in agreement with previous reports^18,43^.

Cells treated with blebbistatin and imaged as above produced numerous thin and highly dynamic protrusions, and exhibited only very low levels of fiber rearrangement (Fig. 7b, Supplementary Movie 7). Many protrusions appeared to follow a meandering path that was not coordinated with neighboring protrusions. Upon closer inspection, we observed that the protrusions were in fact following fibers, generally without regard for orientation (Fig. 7b, i-ii). For example, Fig. 7 bii shows a prominent protrusion that makes a 90° turn (toward the bottom of the image) to follow a string of interconnected fibers. U2OS human osteosarcoma cells, although less protrusive than HT-1080 cells, behaved similarly in separate experiments (Supplementary Fig. S3). Therefore, fibers guide the direction of individual protrusions, even under MII inhibition and even when the fibers are not aligned. Again, had the cell been presented with pre-aligned fibers, we hypothesize that the individual protrusions would have progressed in the same direction, leading to polarized cell migration. Taken together, our experiments in 3D collagen and on 2D fibrillar scaffolds support the hypothesis that the continuous adhesive surface afforded by a fiber is sufficient to enable contact guidance of individual protrusions.

## Discussion

Contact guidance is an important cell migration guidance cue in numerous physiological contexts; however, while this behavior seems intuitive and is frequently observed, its underlying mechanisms are still being elucidated. In particular, it is not understood how contact guidance can occur in the absence of MII organization and activity. This study demonstrates that contact guidance is regulated locally: individual protrusions are guided by local fiber orientation independently of one another and independent of MII activity. Individual protrusion stability and persistence are a function of adhesion lifetime, which depends on fiber orientation. However, when MII activity is inhibited and adhesion lifetime no longer depends on fiber orientation, the ability of protrusions to form closely spaced adhesions sequentially without having to skip over gaps in adhesive area, biases protrusion persistence along fibers. Therefore, while inhibiting MII activity eliminates polarity with regard to where protrusions form, protrusion stability and persistence are maintained in the direction of fiber alignment resulting in the co-alignment of multiple protrusion fronts and cell polarization. If substrate fibers do not present a uniform orientation, contact guidance of individual protrusions still occurs on a local level, but this does not result in an overall cell polarization. These results provide a mechanism for how aligned features can polarize a cell in the absence of MII activity and, more broadly, demonstrate how contact guidance of an entire cell is built on the local alignment of adhesions and individual protrusions.

The role of MII in contact guidance has been tested with numerous cell types, substrates, and inhibitors, with some studies reporting an adverse effect of MII inhibition (e.g. refs^20,21,24,34–37^), while others reporting no effect (e.g. refs^16,23,31–33^). The varied observations may be due in part to differences in experimental design. For example, Ray *et al*.^20^ and Wang and Schneider^37^ noted that more-contractile cells showed a greater response to MII inhibition than less-contractile cells. In addition, the magnitude of the contact guidance effect may depend on substrate type (e.g. grating vs. printed lines vs. aligned collagen fibers)^37^ and substrate design (e.g. grating depth and spacing)^2^. Differences are likely not due to whether contact guidance was evaluated based on cell polarity (morphology) or migration persistence as studies using both metrics found that they generally agreed^20,34,37^. The differences also cannot be easily explained by inhibitor type: some studies have observed differences between the effects of blebbistatin and ROCK inhibition (by Y-27632 or H1152)^37^, while other studies have not^21,31,35^. Nevertheless, it is notable that most of the studies listed here in which MII inhibition had an effect, did not see a reduction in contact guidance to the level of a negative control such as a cell on an isotropic surface^20,21,34,35,37^. Therefore, in these studies, which used varied substrates, cell types, and inhibitors, MII inhibition reduced but did not eliminate contact guidance. For example, Wang and Schneider^37^ showed that Y-27632 reduced the migration persistence of MDA-MB-231 breast cancer cells on gratings with aligned ridges, but not to the level of cells on a planar substrate. They saw reduced levels of contact guidance in less-contractile MTLn3 cells; however again, the levels of these cells, even under Y-27632 inhibition, were still higher than on planar controls^37^. These experiments demonstrate that, although contact guidance is affected by MII activity, it can persist in its absence; findings that are entirely consistent with our results.

Contact guidance is hypothesized to depend in part on substrate feature orientation regulating adhesion maturation by limiting the area for adhesion elongation^7,19–22,24^. In this study we show that this principle extends beyond single adhesions: aligned fibers provide a continuous area for the sequential formation of adhesions in persistent protrusions. Notably, the lifetime of individual adhesions was not necessarily a mitigating factor, since the contact guidance of individual protrusions still occurred in the presence of MII inhibition when adhesion lifetimes on aligned and oblique fibers were similar. Note that, in Figs 3b and 5b, although adhesions do form sequentially, they have relatively short lifetimes with the earliest adhesions not persisting throughout the duration of the movies. Thus it appears that, at least under MII inhibition, the ease of sequential adhesion formation plays a larger role than adhesion lifetime in directing local contact guidance. It is nevertheless possible that physiologically significant differences in adhesion lifetimes on aligned and oblique fibers do exist in MII-inhibited cells, but that they are smaller than the resolution of our analysis. Regardless of whether local protrusion polarity is guided by individual or sequential adhesion dynamics, both are instances of haptotaxis: increased adhesion leads to increased protrusive activity and stability. These results support previous studies of cells on isolated (or pairs of) polymer fibers^23^ and printed lines of fibronectin^30^ showing that narrow aligned features concentrated adhesion formation in defined areas, leading to polarization^23^, higher protrusion rates, and higher adhesion stability compared to cells on isotropic surfaces^30^. However, in contrast to the work presented here, the cells in these studies were confined to one or two features (fibers or lines) and did not have alternative features on which to spread and deviate from the substrate-imposed polarity.

Our results support the theory that, at its most basic level, contact guidance in tissues is a function of adhesion area regulation^7,19–22,24^. While anisotropic mechanical properties likely are involved in many physiological contexts^15,25,26^, contact guidance can occur in their absence and/or under impaired mechanosensitivity (e.g. MII inhibition). Eliminating adhesive area as a guidance cue would require making the substrate structurally isotropic. For example, Autenrieth *et al*.^44^ measured polarized migration of cells on patterns of 2 × 2 µm squares with a gradient in spacing to study haptotaxis. The cells migrated in the direction of decreasing spacing (increasing pattern density), but migrated randomly when MII was inhibited. Because adhesive area was discontinuous in all directions (instead of only one direction as with aligned fibers), the sequential adhesion mechanism identified in our study would not apply; rather the mechanism by which the cells sensed the spacing gradient was MII-dependent.

A more physiologically relevant situation that would eliminate adhesive area as a guidance cue is decreased fiber spacing sufficient to allow adhesions to freely bridge the gaps or to allow sequential adhesion formation. (With regard to sequential adhesions, it is important to remember that the 2 µm average spacing measured in Fig. 5 is likely a substantial overestimate.) Ramirez-San Juan *et al*.^34^ measured NIH 3T3 fibroblast orientation on micropatterened parallel lines with variable spacing and observed a modest decrease in cell alignment with decreasing fiber spacing (from 2 to 10 µm). However, the smallest spacing they tested was 2 µm, which still enabled significant cell alignment. It is likely that much finer features would need to be tested to determine the threshold for contact guidance. For example, numerous cell types have been observed to align to grooves spaced as close as 130 nm (depth, 100 nm)^45^. As fiber spacing decreases, the surface becomes essentially non-fibrillar, which could occur, for example, along blood vessels or if a large number of ECM fibers were tightly bundled. However, even in such cases, cell adhesion would still be limited by the edges of the bundle or vessel.

Importantly, as has been widely shown, cells themselves can generate and modify contact guidance cues by rearranging the fiber structures in their local environment. Here, we show that this begins at the earliest timepoints—at the onset of spreading. Similar to recently published observations^18^, we see that early cell-mediated alignment of collagen fibers guides initial stable cell protrusions and that this cycle of local alignment and protrusion continues as the cell protrusions progress. These findings imply that cells using adhesion-based migration in matrices with flexible fibers are never really sensing randomly oriented fibers; there is always some amount of fiber alignment—even if only local and temporary.

Structured adhesive areas are an inherent property of fibrillar environments and likely affect most aspects of adherent cell-ECM interaction. We demonstrate that this property is important in guiding cell polarity through its hierarchical effects on adhesion and protrusion polarization. Although there are other microenvironment properties that affect migration in 3D environments^2,3,46^ and other modes of migration^47^, adherent cells encountering distinct fibers should be at least partially guided by adhesive area structuring. This has important implications for the design of artificial tissues that seek to promote and therapeutics that seek to inhibit cell migration. For example, inhibiting cellular mechanosensing may not prevent tumor cell invasion when contact guidance cues are present. Indeed squamous cell carcinoma cells were able to invade collagen matrices under Rho or Rho-kinase inhibition when they were preceded by fibroblasts that produced tracks for the cells to follow^48^. Alternatively, immigration of cells into an artificial tissue scaffold may be impaired if the scaffolds fibers are not aligned and not sufficiently flexible to be aligned by migrating cells. Further research into how cells integrate the myriad guidance cues they receive will help us know how to better control cell migration for the remediation of pathologies and the regeneration of tissues.

## Materials and Methods

### Cell culture, transfection, staining, and inhibitors

HT-1080 human fibrosarcoma cells (ATCC, CCL-121) were cultured in MEM + Earl’s Salts, L-glutamine, 10% fetal bovine serum (FBS), and non-essential amino acids. U2OS human osteosarcoma (ATCC, HTB-96) cells were cultured in McCoy’s 5 A medium + 10% FBS. NIH 3T3 mouse fibroblasts (ATCC, CRL-1658) were cultured in high-glucose DMEM + 10% FBS. During experiments, the cells were cultured in CCM1 (a CO_2_-independent medium; Hyclone, Thermo Fisher Scientific). All cell-culture reagents were from Thermo Fisher Scientific unless otherwise indicated.

HT-1080 cells were transfected with TransIT-2020 (Mirus Bio; Madison, WI). The EGFP-paxillin plasmid has been previously described^49^ (Addgene, 15233) Ruby-lifeact (actin probe) was a gift from R. Wedlich-Soldner (Max-Planck Institute of Biochemistry, Germany)^40^.

For timelapse/morphology experiments, U2OS and HT-1080 cells were stained with DiI (Molecular Probes, Thermo Fisher). DiI staining in NIH 3T3 cells was too patchy to allow accurate morphology quanitification; therefore cells were stained with phalloidin-TRITC (Cytoskeleton) for morphology measurements (not timelapse). Briefly, NIH 3T3 samples were fixed with 4% formaldehyde, permeabilized with 0.5% Triton X-100, blocked with 4% BSA, and then stained with a 1:200 dilution of phalloidin-TRITC before being mounted on coverslips.

Inhibitors: Y-27632 (Calbiochem and Cayman Chemical) stock was prepared in deionized water and diluted to a final working concentration of 20 μM in CCM1. ML-7 (Calbiochem) stock was prepared in DMSO and diluted to 10 µM in CCM1. Blebbistatin (+/−) (Calbiochem and Cayman Chemical) stock was prepared in DMSO and diluted to 50 and 100 µM in CCM1.

### 3D collagen sample experiments

3D collagen samples were prepared according to our previously published protocols^50^. Collagen matrices for all experiments consisted of 200 µl 1.2 mg/ml non-pepsinized rat-tail collagen (Gibco, Thermo Fisher) in CCM1 medium with 30 × 10^3^ cells per matrix and inhibitors as necessary. Samples were quickly moved to the microscope and imaging began within 15 minutes. A dish heater mounted on the microscope stage maintained the samples at 37 °C and ensured that gelation of the collagen matrix continued normally. Z-stacks were acquired every 3 min for 30–40 min.

### PCL scaffold experiments

20 µm thick scaffolds with random or aligned 700 nm diameter electrospun polycaprolactone (PCL) fibers, mounted on 15 mm diameter plastic coverslips were purchased from Nanofiber Solutions (NanoAligned and NanoECM 24-well plate inserts, respectively). The scaffolds were placed in 12-well plates and adsorbed with 5 µg/ml human plasma fibronectin (Invitrogen and Corning, Fisher Scientific) in PBS for 30 min at room temperature or overnight at 5 °C. The scaffolds were rinsed once with PBS before seeding cells.

For timelapse experiments, cells were seeded on scaffolds in CCM1 (containing inhibitors as necessary) and then incubated for 10 min at 37 °C. The scaffolds were then inverted, placed in a glass-bottomed dish (produced in-house) filled with CCM1 (containing inhibitors as necessary), and quickly moved to the microscope where imaging began with 20 min of cell seeding. Z-stacks for high-resolution adhesion timelapse movies were acquired every 2 min for 20–60 min. Z-stacks for low-magnification, multi-cell movies were acquired every 1 min for 30–45 min.

For morphology experiments, cells were seeded on scaffolds in CCM1 (containing inhibitors as necessary), incubated for 30 min at 37 °C, fixed with 4% formaldehyde for 15 min, and then mounted on coverslips.

### Basic imaging protocol and image processing

Except as indicated below, samples were imaged on an Olympus Fluoview 1000 laser scanning confocal microscope with a UPlanSApo 60x (1.20 NA) water-immersion objective. Settings were adjusted to give pixel dimensions of 0.08–0.10 µm in the x-y direction and 0.33 µm in the z-direction. Low-magnification, multi-cell movies on PCL scaffolds were acquired with a UPlanFluor 10x (0.30 NA) objective and x-y pixel dimensions of 0.83 µm. Samples were excited with the appropriate laser lines: 488 nm Ar-ion laser (for GFP) and 543 nm HeNe laser (for Ruby and DiI). Collagen and PCL fibers were imaged simultaneously by confocal reflectance microscopy. Settings were adjusted to minimize photodamage. Some of the HT-1080 and all of the NIH 3T3 morphological data (Fig. 1 and Supplementary Fig. 1) were acquired on a Nikon TE2000 microscope with a PlanApo 20x (0.75 NA) objective and a Photometrics CoolSnap HQ2 monochrome camera. DiI and TRITC-phalloidin fluorescence was excited by a mercury arc lamp (X-Cite, Excelitas) and collected using a standard long-pass filter set (Ex: 545/22, Em: 605/70). PCL fiber images were acquired using transmitted light. Live samples were maintained at 37 °C with a stage-mounted dish heater (Warner Instruments). Sample pH was maintained using a CO_2_-independent medium (CCM1, see above).

Images and videos were processed and analyzed with MATLAB (MathWorks) and ImageJ (http://rsb.info.nih.gov/ij/). Unless otherwise indicated, the intensities of images in the displayed figures and movies were not modified except for linear adjustments to the display range. Adhesion images in Figs 2, 3, and 5 were “flattened” to reduce background noise. Briefly, individual images were median filtered using a sliding box (20 × 20 pixels) filter, then the filtered image was subtracted from the original image to produce a “flattened” image.

### Cell shape and orientation analyses

The cell shape analysis procedure has been previously described^22^. Briefly, all protrusive cells with sufficient fluorescent intensity within a given area of each sample were imaged. Images were acquired at a resolution of 300–400 nm/pixel, which allowed fast acquisition and processing, but was still sufficient to identify non-filopodia protrusions. Cell “circularity” and orientation were measured with custom-written, semi-automated MATLAB scripts. Images were subjected to interactive thresholding, resulting in a binary image (a 2D silhouette). The circularity (c) was calculated as c = 4πA/P^2^, where A is the area and P is the perimeter. Cell area, perimeter, and orientation were determined by pre-packaged MATLAB algorithms.

The distribution of fiber orientations in images of aligned PCL fibers was determined using a custom-written MATLAB script implementing a previously published gradient mask convolution algorithm^51,52^. The mode of the orientation distribution generated by this script was taken as the predominant fiber orientation in the image.

### Adhesion, protrusion, and fiber orientation analyses

Movies of HT-1080 cells migrating on aligned PCL scaffolds and expressing EGFP-paxillin and Ruby-Lifeact were analyzed for correlations between adhesion lifetime, fiber angle, and protrusion fate. Individual adhesions were tracked over time by hand using “flattened” images (see Basic Imaging Protocol and Image Processing, above). Only adhesions that appeared during the course of the movie were analyzed because the lifetimes of adhesions that existed before the beginning of the movie could not be determined. Adhesions that did not disappear before the end of the movie (thus preventing an accurate lifetime measurement) were “censored” during the survival analysis. The protrusion associated with each adhesion was classified as persistent if it actively protruded after formation of the adhesion, retracted if it retracted after adhesion formation, or stable if it did not retract or protrude after adhesion formation. The angle between the direction of protrusion and the fiber on which the adhesion formed was classified as either aligned (~0° angle difference; parallel) or oblique (~90° difference; perpendicular). Nearly all protrusions fell into one of these two categories; protrusions that intersected fibers at intermediary angles were omitted from the analysis.

### Adhesion and fiber spacing analysis

Distances between sequentially formed adhesions were measured only from experiments in which cells were inhibited with Y-27632 and ML-7. Adhesions were considered sequential if they formed in close temporal succession (within 1–2 frames or 2–4 min) and were co-linear (part of the same protrusion and forming in the same direction). Sequential adhesions that formed too closer than 0.5 µm were excluded because distances could not be accurately determined. Measurements were performed using the profiler function in ImageJ on flattened images that were additionally filtered with a sliding block (3 × 3 pixel) Gaussian filter to reduce background noise. Since adhesions did not elongate and remained punctate, distances were measured between adhesion centers (peaks of the intensity profiles). Distances between fibers were measured from the same movies as used for adhesion spacing measurements. Spacing was measured perpendicular to the direction of fiber alignment using the profiler function in ImageJ on maximum-intensity-z-projections of fiber images. Spacing measurements for adhesions and fibers were rounded to the nearest 0.5 µm. The optical resolution of the images was diffraction-limited (approximately 220 nm); the sampling resolution (pixel size) varied from 82 to 98 nm per pixel.

### Statistics

Non-normally distributed data are displayed with box-and-whisker plots: error bars show the 10^th^ and 90^th^ percentiles; lower and upper sides of the box show the 25^th^ and 75^th^ percentiles, respectively; and the line within the box shows the median. The Kruskal-Wallis test was used to test for differences between non-normally distributed populations. Statistics were computed with pre-packaged MATLAB algorithms except for the survival analysis, which was performed using the kmplot.m and logrank.m functions written by G. Cardillo (2008) and downloaded from http://www.mathworks.com/matlabcentral/fileexchange/2293 and /22317, respectively. These two functions use the Kaplan-Meier method and the Log-rank test. Pair-wise comparisons of log-rank data were performed using the sequential Dunn-Sidak method. Graphs were made in SigmaPlot.

### Data Availability

The datasets generated during the current study are available from the corresponding author on reasonable request.

## Electronic supplementary material

Supplementary Movie 1

Supplementary Movie 2

Supplementary Movie 3

Supplementary Movie 4

Supplementary Movie 5

Supplementary Movie 6

Supplementary Movie 7

Supplementary Figures
