# Supplementary material for: Contact guidance persists under myosin inhibition due to the local alignment of adhesions and individual protrusions
Source: Sci Rep. 2017 Oct 30;7:14380. doi: 10.1038/s41598-017-14745-7 (PMC5662575; doi:10.1038/s41598-017-14745-7)
Supplement: Supplementary file 8 — Supplementary Figures [file 41598_2017_14745_MOESM8_ESM.pdf]

Supplementary Information for

Contact guidance persists under myosin inhibition due to the local alignment of adhesions and individual protrusions

Kristopher E. Kubow, Victoria D. Shuklis, Dominic J. Sales, and A. Rick Horwitz

Contents

Page 1 Supplementary Figure S1
Page 2 Supplementary Figure S2
Page 3 Supplementary Figure S3
Pages 4-5 Supplementary Movie captions

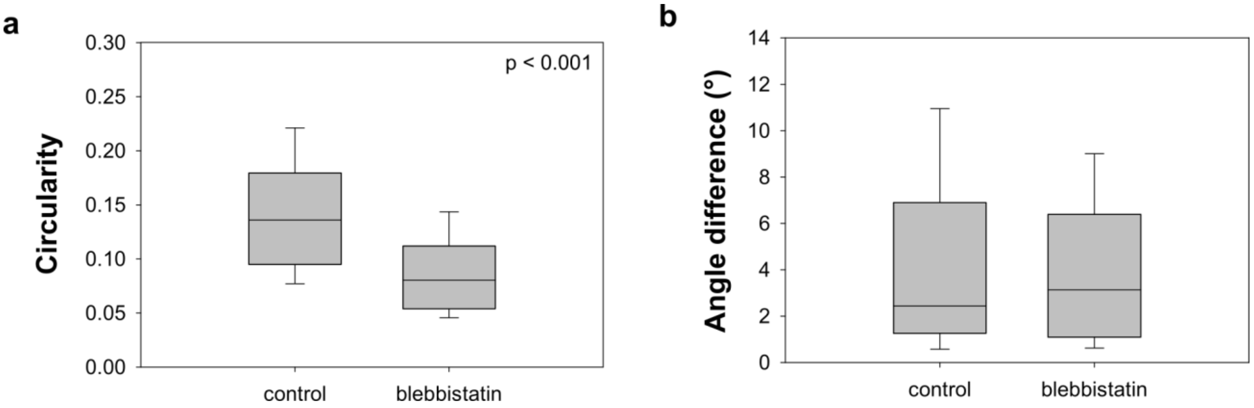

Supplementary Figure S1: NIH3T3 fibroblasts orient to aligned fibers even under MII activity and organization inhibition. Supplementary Figure for Fig. 1. NIH3T3 mouse fibroblast cells, were seeded on 2D PCL scaffolds with aligned fibers, cultured in the presence or absence of 50 µM blebbistatin for 30 min, fixed and stained with fluorescently labeled phalloidin, and then imaged. Cell morphology and orientation were analyzed as in Fig. 1. **(A)** Plot of the "circularity" morphological parameter. Center line of each box indicates the median; upper and lower bounds of the box indicate the 75th and 25th percentiles, respectively; the "whiskers" indicate the 10th and 90th percentiles. Inhibited cells had a significantly lower circularity than control cells ($p < 0.001$, Kruskal-Wallis test), indicating that they exhibited a higher number of protrusions. **(B)** Plot of the difference in angle between cell orientation and the average direction of fiber alignment. All groups showed a median deviation of less than 5 degrees and a deviation of less than 12 degrees for at least 90% of their individuals. Both plots were derived from the same data: 33 cells from control samples, 36 cells from blebbistatin samples, taken from three independent experiments.

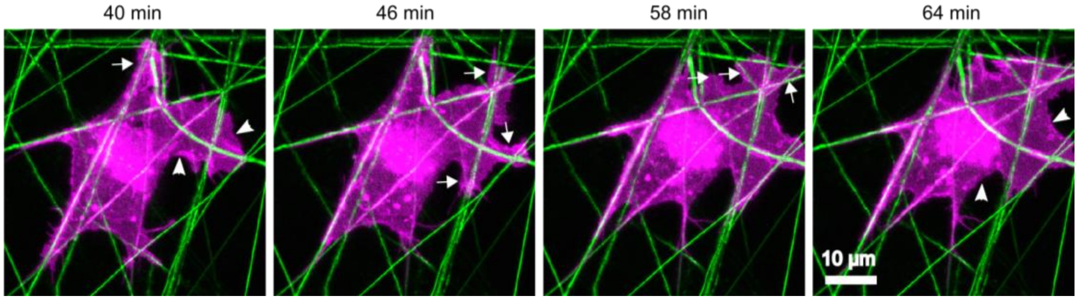

Supplementary Figure S2: HT-1080 cell protrusions exhibit contact guidance along randomly oriented PCL scaffold fibers. Supplementary Figure for Fig. 6. HT-1080 cells, stained with the membrane dye DiI (magenta) were seeded on 2D PCL scaffolds (green) in the presence of 50 µM blebbistatin and imaged over time. Images are from a different experiment than the images in Fig. 6 and are representative of six independent experiments. Times indicated in the figure are relative to the time of seeding. The cell protrudes in multiple directions, with each protrusion independently extending along a fiber (arrows), rather than skipping over gaps between fibers. Lamellae often stretch between protrusions that are anchored to nearby fibers (arrowheads). All images are maximum projections of z-stacks.

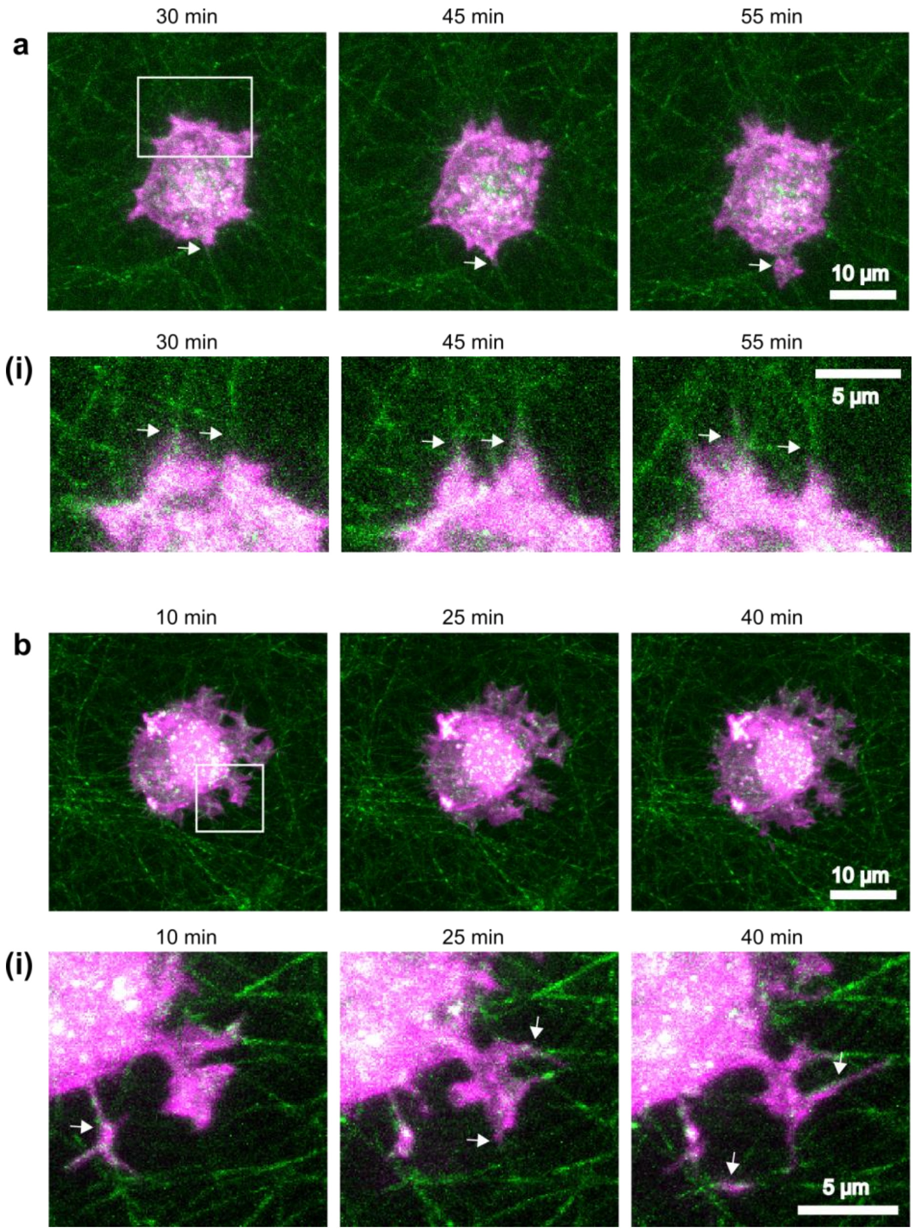

Supplementary Figure S3: U2OS cell protrusions exhibit contact guidance along collagen I fibers under control or blebbistatin-treated conditions. Supplementary Figure for Fig. 7. U2OS cells, stained with the membrane dye DiI (magenta) were seeded in 1.2 mg/ml rat-tail collagen ECMs (green) and imaged over time. Times indicated in the figure are relative to the time of seeding. Primary panels (A and B) are maximum projections of z-stacks. The sub-panels (i) show selected frames and z-slices from regions of interest in the associate full-cell image. **(A)** Selected frames, representative of three independent experiments. U2OS cells are less protrusive than HT-1080 cells; nevertheless, similar to Fig. 7, stable protrusions coincide with centripetally aligned fibers (arrows in main panel and in sub-panel, i). **(B)** Selected frames of a cell treated with 50 µM blebbistatin (representative of three independent experiments). The cell extends numerous highly dynamic protrusions, which are not necessarily localized to areas of high matrix alignment. There is little cell-mediated fiber movement; instead, protrusions often follow fibers like tracks, even if they do not follow a straight line (arrows in sub-panel, i).

SUPPLEMENTARY MOVIE CAPTIONS

Supplementary Movie 1: HT-1080 cells orient to aligned fibers. Corresponds to Fig. 1a. HT-1080 cells, stained with the membrane dye DiI were seeded on 2D PCL scaffolds with aligned fibers. Cells were imaged during spreading and initial migration under control conditions. Cells (DiI stain) are displayed using an intensity-based heatmap to better visualize them in spite of their large differences in brightness. The color of the heatmap has no physiological relevance. The area in the white box corresponds to the frames shown in Fig. 1a. The cells protruded along the fibers, resulting in overall cell alignment to the fibers. Although protrusions perpendicular to the direction of fiber alignment did occur (often during spreading), the protrusions subsequently turned and proceeded along the fibers. Images were acquired every minute, beginning 12 min after seeding.

Supplementary Movie 2: HT-1080 cells orient to aligned fibers even under MII inhibition. Corresponds to Fig. 1b. HT-1080 cells, stained with the membrane dye DiI were seeded on 2D PCL scaffolds with aligned fibers. Cells were imaged during spreading and initial migration in the presence of 20 µM Y-27632 and 10 µM ML-7. Cells (DiI stain) are displayed using an intensity-based heatmap to better visualize them in spite of their large differences in brightness. The color of the heatmap has no physiological relevance. The area in the white box corresponds to the frames shown in Fig. 1b. The cells protruded along the fibers, resulting in overall cell alignment to the fibers. Although cells were more protrusive than control cells and did protrude perpendicular to the direction of fiber alignment, such protrusions subsequently turned and proceeded along the fibers. Images were acquired every minute, beginning 10 min after seeding.

Supplementary Movie 3: The persistence of individual protrusions is correlated with adhesion stability and fiber orientation. Corresponds to Fig. 2. HT-1080 cells expressing EGFP-paxillin (green) and Ruby-Lifeact (actin; magenta) were seeded on 2D PCL scaffolds with aligned fibers (blue) and imaged over time. Note that small fluctuations in focus changed the intensity of the reflectance signal coming from the fibers, resulting in a few fibers appearing very dim in some frames. Frames are maximum projections of z-stacks. The cell forms adhesions primarily along aligned fibers but also in protrusions perpendicular to the direction of alignment. See Fig. 2 for details on specific events occurring in this movie. Z-stacks were acquired every 2 min, beginning 17 min after seeding.

Supplementary Movie 4: The persistence of individual protrusions is correlated with fiber orientation even when under MII inhibition. Corresponds to Fig. 3. HT-1080 cells expressing EGFP-paxillin (green) and Ruby-Lifeact (actin; magenta) were seeded on 2D PCL scaffolds with aligned fibers (blue) in the presence of 20 µM Y-27632 and 10 µM ML-7 and imaged over time. All images are maximum projections of z-stacks. The cell aligns to the predominant direction of fiber orientation, despite it being highly protrusive in nearly all directions. Adhesions (green) are generally small. The cell forms a large protrusion perpendicular to the direction of fiber alignment near the beginning of the movie, then turns approximately 90 degrees and protrudes along the aligned fibers, progressively forming small transient adhesions as it extends. See Fig. 3 for details on specific events occurring in this movie. Z-stacks were acquired every 2 min, beginning 18 min after seeding.

Supplementary Movie 5: HT-1080 cell protrusions exhibit contact guidance along randomly oriented PCL scaffold fibers. Corresponds to Fig. 6. HT-1080 cells, stained with the membrane dye DiI (magenta) were seeded on 2D PCL scaffolds (green) in the presence of 50 µM blebbistatin and imaged over time. The cell is in the process of spreading at the beginning of imaging. Protrusions extend along fibers, often with lamellae stretching between them. See Fig. 6 for details on specific events occurring in this movie. All images are maximum projections of z-stacks. Z-stacks were acquired every 2 min, beginning 10 min after seeding.

Supplementary Movie 6: HT-1080 cell protrusions exhibit contact guidance along collagen I fibers. Corresponds to Fig. 7a. HT-1080 cells, stained with the membrane dye DiI (magenta) were seeded in 1.2 mg/ml rat-tail collagen ECMs (green) and imaged over time. Frames are maximum projections of z-stacks. Initially, the cell initially protrudes in multiple directions and stable protrusions coincide with centripetally aligned fibers. Next, the cell begins moving predominately in one direction (toward the bottom of the frame) and the associated protrusion/retraction activity compacts fibers and aligns them in the direction of migration. See Fig. 7a for a detailed description of events occurring in this movie. Z-stacks were acquired every 3 min, beginning 7 min after seeding.

Supplementary Movie 7: HT-1080 cell protrusions exhibit contact guidance along collagen I fibers under MII inhibition. Corresponds to Fig. 7b. HT-1080 cells, stained with the membrane dye DiI (magenta) were seeded in 1.2 mg/ml rat-tail collagen ECMs (green) in 50 µM blebbistatin and imaged over time. Frames are maximum projections of z-stacks. The cell extends many thin and highly dynamic protrusions. Protrusions are not localized to areas of high matrix alignment, but a higher number of protrusions extend toward the bottom of the frame, possibly in response to the predominant alignment of the fibers. There is little cell-mediated fiber movement; instead, protrusions often follow fibers like tracks, even if they do not follow a straight line. See Fig. 7b for a detailed description of events occurring in this movie. Z-stacks were acquired every 3 min, beginning 6 min after seeding.
